# Lumikine Attenuates Ventilation-Induced Lung Injury in Mice with Lipopolysaccharide-Induced Lung Fibrosis

**DOI:** 10.3390/ijms27198515

**Published:** 2026-09-24

**Authors:** Li-Fu Li, Pao-Hsien Chu, Winston W.-Y. Kao, Chung-Chieh Yu, Chih-Yu Huang, Huang-Pin Wu, Chien-Ming Chu, Ping-Chi Liu, Yung-Yang Liu

**Affiliations:** 1 Division of Pulmonary and Critical Care Medicine, Department of Internal Medicine, Chang Gung Memorial Hospital, Keelung 20401, Taiwan; lfp3434@cgmh.org.tw (L.-F.L.); ycc@cgmh.org.tw (C.-C.Y.); hcu121@yahoo.com.tw (C.-Y.H.); whanpyng@cgmh.org.tw (H.-P.W.); rocephen@cgmh.org.tw (C.-M.C.); ewind14@cgmh.org.tw (P.-C.L.); 2 Department of Internal Medicine, Chang Gung University, Taoyuan 33302, Taiwan; 3 Department of Respiratory Therapy, Chang Gung Memorial Hospital, Keelung 20401, Taiwan; 4 Community Medicine Research Center, Chang Gung Memorial Hospital, Keelung 20401, Taiwan; 5 Division of Cardiology, Department of Internal Medicine, Chang Gung Memorial Hospital, Chang Gung University College of Medicine, Taoyuan 33302, Taiwan; taipei.chu@gmail.com; 6 Institute of Stem Cell and Translational Cancer Research, Chang Gung Memorial Hospital, Chang Gung University College of Medicine, Taoyuan 33302, Taiwan; 7 Department of Ophthalmology, University of Cincinnati, Cincinnati, OH 45267, USA; kaoww@ucmail.uc.edu; 8 Chest Department, Taipei Veterans General Hospital, Taipei 112201, Taiwan; 9 School of Medicine, College of Medicine, National Yang Ming Chiao Tung University, Taipei 112304, Taiwan; 10 Institute of Clinical Medicine, School of Medicine, National Yang Ming Chiao Tung University, Taipei 112304, Taiwan

**Keywords:** acute lung injury, pulmonary fibrosis, lumican, ventilator-induced lung injury

## Abstract

Lipopolysaccharide (LPS) induces pulmonary fibrosis by increasing microvascular permeability and inducing severe inflammation and extracellular matrix accumulation. Mechanical ventilation (MV), although life-sustaining, can increase inflammatory cytokine production, oxidative stress, and alveolar–capillary membrane permeability, leading to ventilator-induced lung injury and pulmonary fibrosis. Lumican is essential for extracellular matrix signaling and wound fibrogenesis in the lungs, cornea, and heart. Lumikine, a peptide corresponding to the 13 C-terminal amino acids of lumican, binds to transforming growth factor-β receptor I/activin receptor-like kinase 5 and promotes wound healing. How the lumican pathway contributes to MV-induced lung inflammation and fibrosis remains unclear. We hypothesized that MV with or without LPS pretreatment would exacerbate lung injury, oxidative stress, and lung fibrosis through the lumican pathway. C57BL/6 mice (wild-type and lumican-deficient) were injected with LPS (intratracheal; 7 days) to induce lung fibrosis and then subjected to MV (10 mL/kg; room air; 8 h). Nonventilated mice served as controls. MV with LPS pretreatment reduced lumican expression and increased microvascular permeability, matrix metalloproteinase-9 level, transforming growth factor-β1 level, α-smooth muscle actin staining intensity, and Masson’s trichrome staining intensity. Lumikine substantially mitigated these fibrotic changes. In conclusion, lumikine attenuates MV-induced exacerbation of pulmonary fibrosis after LPS-induced acute lung injury, partially by modulating the lumican pathway.

## 1. Introduction

Prior murine studies on acute lung injury (ALI) have reported that lipopolysaccharide (LPS) induces rapid pulmonary fibrosis when directly administered into lung tissue [1,2]. LPS-induced lung injury initially increases microvascular permeability and capillary leakage by disrupting epithelial and endothelial barrier integrity, thereby eliciting a severe inflammatory response [1,2,3]. The resulting pulmonary inflammation is followed by a reparative phase characterized by the accumulation of the extracellular matrix (ECM) [4,5]. Chronic ventilator dependence or mortality after ALI is often a consequence of abnormal wound repair, which is characterized by severe hypoxemia, extensive fibrosis, and loss of lung compliance [6]. Mechanical ventilation (MV) is commonly used to provide respiratory support to patients with ALI. However, prolonged MV may increase the risk of pathological lung stretch, recruitment of inflammatory cells, and production of inflammatory mediators, leading to a form of ALI known as ventilator-induced lung injury (VILI). Three major factors drive the differentiation of fibroblasts to myofibroblasts: increases in mechanical stress, local upregulation of matrix metalloproteinase-9 (MMP-9) and transforming growth factor-β1 (TGF-β1), and presence of the extra domain A splice variant of fibronectin [7]. Aberrant mechanical stretch can disrupt cellular barrier function and induce cytotoxicity in the lungs of patients with ALI [8]. The mechanisms that determine whether lung injury resolves or progresses to advanced fibrosis remain unclear.

Small leucine-rich proteoglycans, including lumican, interact with various cell membrane receptors, inflammatory cytokines, and ECM molecules to regulate cellular processes such as epithelial cell migration, fibroblast proliferation and apoptosis, and collagen fibril organization [9]. Lumican is a major component of adult human lung tissue, whereas other small leucine-rich proteoglycans (e.g., decorin, biglycan, and fibromodulin) are less abundant [10]. In the cornea, lumican regulates collagen fibrogenesis and collagen fiber organization, which are essential for maintaining optical clarity. Accordingly, lumican knockout mice exhibit severe disturbances in ECM organization and loss of corneal transparency [3,11]. Lumican contributes to ECM assembly in the lungs, which is essential for maintaining the mechanical stability and elastic recoil required for normal lung function. In a murine model of pulmonary arterial hypertension, lumican deficiency promoted pulmonary arterial remodeling [12]. Lumikine was demonstrated to reverse the pathological changes associated with hypoxia-induced pulmonary arterial hypertension [12]. Together, these findings suggest that lumican plays a complex role in regulating inflammation and fibrosis and can be a therapeutic target in lung injury and fibrotic diseases. However, the mechanisms through which lumican contributes to fibrogenesis in VILI remain unclear.

Lumikine (LumC13C-A), a peptide corresponding to the 13 C-terminal amino acids of lumican, binds to TGF-β receptor I/activin receptor-like kinase 5, forming a stable complex and promoting wound healing from earlier reports [12,13]. It promotes corneal epithelial cell migration and wound healing both in vitro and in vivo [14]. However, the mechanisms underlying the interactions among MV, lung fibrogenesis, and lumican expression remain unclear. Identifying the mechanisms that regulate lung injury can facilitate the development of therapeutic strategies for pulmonary fibrosis after ALI.

In the present study, we used a murine model of LPS-induced VILI with fibrosis to investigate the mechanisms underlying the fibroproliferative phase of ALI. The objectives were as follows: (1) to assess changes in lumican expression due to the development of pulmonary fibrogenesis during MV, (2) to compare inflammatory cytokine and oxidative stress levels between MV-induced lung injury and LPS-induced lung injury, and (3) to identify the role of lumican in VILI by using lumikine. We hypothesized that MV, with or without LPS pretreatment, would induce lung inflammation, epithelial–mesenchymal transition (EMT), increased free radical production, and pulmonary fibrogenesis by regulating the lumican pathway.

## 2. Results

### 2.1. Lumikine Attenuated LPS-Enhanced MV-Induced Microvascular Leakage, Oxidative Stress, MMP-9, and TGF-β1 Production

We used MV (tidal volume [V_T_] = 10 mL/kg) with room air for 8 h to induce VILI in mice and to examine the effects of intraperitoneally administered lumikine, a lumican-derived peptide [12]. The physiological parameters measured at the beginning and end of MV are presented in Table S1. Euvolemia was maintained throughout the experiment, as confirmed through the monitoring of mean arterial pressure. Changes in MV-induced microvascular permeability were examined by measuring Evans blue dye extravasation in the lungs (Figure 1A). Levels of inflammatory cytokines and oxidative stress markers were measured to evaluate pulmonary inflammation and oxidative stress in VILI (Figure 1B–E). Compared with mice subjected to V_T_ = 10 mL/kg without LPS pretreatment and nonventilated control mice, LPS-treated mice subjected to V_T_ = 10 mL/kg exhibited increased levels of Evans blue dye, malondialdehyde (an aldehydic secondary product of lipid peroxidation), MMP-9, and TGF-β1 and reduced the production of superoxide dismutase (Figure 1). Lumikine substantially attenuated lung injury in LPS-treated mice subjected to MV (Figure 1).

### 2.2. Lumikine Blocked the Effects of MV on LPS-Mediated Collagen Fiber Formation and Fibrogenesis

Masson’s trichrome staining was performed to examine the effects of MV on collagen fiber deposition in the peribronchiolar and parenchymal regions of the lungs (Figure 2A). Collagen fibers were more abundant in the ECM of LPS-treated mice subjected to a V_T_ of 10 mL/kg than in the ECM of mice subjected to a V_T_ of 10 mL/kg without LPS pretreatment and in that of nonventilated control mice (Figure 2A). We performed micro-computed tomography (CT), a primary imaging modality used for the clinical assessment of pulmonary fibrosis, to examine structural lung damage (Figure 2B). Pulmonary fibrotic changes, such as increased reticular opacity, honeycombing, and ground-glass opacity in the lower lungs, were more pronounced in LPS-treated mice subjected to a V_T_ of 10 mL/kg than in mice subjected to a V_T_ of 10 mL/kg without LPS pretreatment and in nonventilated control mice, as indicated by a densitometric analysis of Hounsfield units in the lung parenchyma (Figure 2B). Furthermore, the expression of zonula occludens-1 (ZO-1; epithelial marker) and α-smooth muscle actin (α-SMA; mesenchymal marker) was analyzed using immunofluorescence staining to evaluate EMT induced by lung stretch (Figure 3A,B). In addition, total lung collagen content and fibrosis scores were obtained using Masson’s trichrome staining to determine the effect of MV on ECM deposition (Figure 3C,D). Lumikine substantially attenuated the LPS-enhanced MV-induced increase in collagen fiber deposition, downregulation of ZO-1, and upregulation of α-SMA (Figure 3).

### 2.3. Lumikine Attenuated the Effect of MV on LPS-Induced Lumican Expression

Because lumican activation modulated LPS-mediated cardiac and corneal fibrosis, we evaluated lumican expression to determine the role of the lumican pathway in VILI-associated pulmonary fibrosis (Figure 4) [12,13]. Western blotting revealed lower lumican expression in mice treated with LPS and MV with room air than in nonventilated control mice (Figure 4). Lumikine substantially attenuated the reduction in lumican expression observed in mice treated with LPS and MV (Figure 4). Immunohistochemistry was performed to identify cell types involved in mechanical stretch-induced fibrogenesis (Figure 4C). Immunohistochemical staining revealed a marked downregulation of lumican in the airway epithelial cells of mice treated with LPS and MV compared with the expression levels in nonventilated control mice (Figure 4C). Consistent with the Western blotting results, lumikine blocked the LPS-enhanced MV-induced reduction in lumican expression (Figure 4).

### 2.4. Lumican-Deficient Mice Exhibited No Attenuation of the Effects of MV on LPS-Induced Lung Inflammation and EMT

Lumican-deficient mice were used to determine whether the beneficial effects of lumikine were mediated through the lumican pathway. In lumican-deficient mice treated with LPS and MV, the effects of MV on microvascular permeability, inflammatory cytokine production, oxidative stress, collagen fiber accumulation, fibrogenic marker expression, and fibrosis scores, were not attenuated (*p* < 0.05; Figure 5 and Figure 6). These findings suggest the protective effects of lumikine against MV- and LPS-induced lung inflammation and fibrosis are mediated through the lumican pathway (Figure 7).

## 3. Discussion

Sepsis is a severe, life-threatening condition characterized by a dysregulated host response to infection and is associated with ALI and multiple organ dysfunction. Notably, sepsis-associated ALI resulting from direct lung injury primarily affects the alveolar epithelium and is characterized by intense pulmonary inflammation. This form of ALI is associated with relatively severe lung injury, increased pulmonary vascular permeability, and reduced lung compliance. Thus, management often requires positive-pressure MV to maintain adequate ventilation and oxygenation [14,15]. Pulmonary dysfunction is common in patients with sepsis and is associated with increased mortality, particularly in those who develop acute respiratory distress syndrome (ARDS) [16].

The fibroproliferative response that follows acute exudative inflammation has previously been regarded as a characteristic of late ARDS and is characterized by fibroblast infiltration, ECM deposition, and collagen accumulation in injured lung tissue. However, clinical evidence indicates that enhanced fibroproliferative activity begins within 24 h of ARDS onset [17,18]. Pulmonary fibrosis contributes to ventilator dependence, restrictive lung dysfunction, and increased mortality, resulting in poor short- and long-term clinical outcomes [12,13]. Actually, survivors of sepsis-associated ALI often experience persistent alterations in respiratory control and cardiorespiratory function that extend beyond the acute inflammatory and fibrotic responses [19,20,21]. Thus, novel pharmacological treatments for sepsis-associated post-ALI pulmonary fibrosis are urgently required to improve clinical outcomes and reduce functional impairment.

The present study demonstrates that the synthetic lumican peptide lumikine can effectively (1) mitigate alveolar–capillary barrier leakage and oxidative stress and restore antioxidant capacity; (2) reduce MMP-9 (inflammatory mediator) level, TGF-β1 (profibrogenic mediator) level, and total collagen deposition; (3) increase lumican expression and deposition in the bronchiolar epithelium; (4) attenuate EMT; (5) suppress lung fibrosis, as evidenced by improvements in Masson’s trichrome staining scores, pathological fibrosis scores, and micro-CT results indicating lower lung attenuation in mice with MV-exacerbated LPS-induced pulmonary fibrogenesis. As expected, lumican-deficient (Lum^−/−^) mice did not exhibit the beneficial effects of lumikine noted in our animal model of lung fibrosis, which was designed to specifically investigate how pre-existing fibrotic remodeling alters susceptibility to VILI, rather than to directly replicate human ARDS pathogenesis. This study allows exploration of how lumikine modifies VILI in the context of fibroproliferative lung pathology, rather than acute inflammation alone. Notably, we demonstrate that lumkine confers protection in a fibroproliferative setting, which differs from both our prior work and that of Lai et al. [3,12].

LPS engagement with Toll-like receptor 4 (TLR4) rapidly activates NADPH oxidases, resulting in excessive reactive oxygen species (ROS) [22]. ROS act as upstream signals that activate NF-κB and MAPK pathways, driving the transcription of pro-inflammatory cytokines and chemokines. Infiltrating neutrophils and macrophages further amplify oxidative stress and secrete proteolytic enzymes, notably MMP-9 [23]. Sustained inflammatory signaling elevates TGF-β1 and EMT-associated transcription factors, while MMP-9 contributes to degradation of ECM components such as lumican and E-cadherin, thereby facilitating EMT [24]. MV and LPS likely contribute to lung injury through overlapping but distinct mechanisms [2,3,16,22]. MV causes biophysical injury through volutrauma, mechanical stretch, cyclic stress, endothelial injury, and epithelial junction disruption, whereas LPS predisposes the lungs to injury through innate immune activation, neutrophil and macrophage recruitment, cytokine release, and oxidative stress. VILI results from interactions among mechanical forces, immune activation, and metabolic and oxidative stress rather than from pressure–volume injury alone [3]. The combination of LPS and MV represents a clinically relevant two-hit model in which endotoxemia primes innate immune and oxidative pathways, whereas excessive lung stretch superimposes mechanical stress on an already vulnerable alveolar–capillary barrier. In the present study, both LPS and MV increased the production of malondialdehyde, MMP-9, TGF-β1, and total collagen compared with corresponding levels in control cells. The combination of LPS and MV exacerbated lung inflammation and oxidative injury. Notably, lumikine–lumican signaling primarily suppresses oxidative injury first, and the secondary inflammation and fibrogenesis in mice with MV-exacerbated LPS-induced sepsis.

EMT is a postinjury tissue repair process during which alveolar epithelial cells lose their epithelial characteristics and acquire mesenchymal features. This process is accompanied by morphological transformation, cellular polarity loss, changes in secretory phenotype, and cytoskeletal reorganization [25]. Activation of EMT promotes the production and deposition of fibrillar collagens and other ECM components, thereby contributing to pathological fibrogenesis [7]. In the present study, lumikine substantially reduced pulmonary oxidative stress, inflammation, α-SMA expression, ZO-1 disruption, and fibrogenesis induced by LPS plus MV. We used micro-CT to assess changes in pulmonary fibrogenesis because densitometric analysis provides quantitative information that enables comparisons among experimental groups. A clinical study reported that quantitative CT–based assessment of lung fibroproliferation severity can yield prognostic data for the evaluation of ventilator dependence and functional outcomes [18]. In addition to biomolecular and histopathological findings, we provided radiological evidence of improved lung attenuation on micro-CT after lumikine treatment in our murine model of pulmonary fibrosis.

Lumican is a key component of the ECM in various tissues, including the mature human lungs [26]. Induction of lumican expression in bronchial epithelial cells results in ECM-associated airway remodeling during allergic airway inflammation in children with asthma [27]. Lumican functions as a crucial endogenous modulator of the TGF-β pathway and EMT [28]. It plays a pivotal role in maintaining corneal transparency by regulating collagen fibrillogenesis during wound healing, epithelial cell migration, and EMT in the injured lens [29]. Adenovirus-mediated lumican was demonstrated to ameliorate hypertrophic scarring by upregulating lumican expression, reducing fibroblast proliferation, and inhibiting ECM accumulation through the suppression of integrin-mediated focal adhesion kinase signaling [30].

Lumican plays a key role in the regulation of pathological pulmonary vascular remodeling associated with pulmonary arterial hypertension. Synthetic lumikine suppresses Akt phosphorylation and inhibits pulmonary arterial smooth muscle remodeling, whereas lumican deficiency promotes pulmonary arterial remodeling [12]. Engebretsen et al. demonstrated markedly increased lumican levels in the myocardium of mice with aortic banding–induced heart failure and that of patients with end-stage heart failure, implicating lumican in the pathophysiology of cardiac remodeling and fibrosis [31]. In a subsequent study, the researchers reported that lumican-deficient mice had a substantially higher mortality rate between days 1 and 14 after pressure overload than did mice with intact lumican expression. The surviving lumican-deficient mice exhibited more severe cardiac chamber dilation and impaired systolic function [32]. These findings suggest that increased lumican expression in heart failure is a compensatory protective response rather than a pathogenic mechanism that drives disease progression. Upregulation of lumican may help the heart counteract mechanical stress and maintain structural integrity under conditions of pressure overload. However, as heart failure progresses, downregulation of lumican expression or depletion of lumican may contribute to irreversible cardiac remodeling and fibrosis.

As shown in Figure 4, lumican expression was increased in the MV-treated group. This finding is consistent with those of our previous animal studies on ALI, in which high-V_T_ MV induced lung and diaphragm injury, activated TGF-β1 signaling, and induced EMT by upregulating lumican [3,33]. The detrimental effects mediated through lumican activation in VILI and ventilator-induced diaphragmatic dysfunction were attenuated in lumican-deficient (Lum^−/−^) mice [3,33]. Patients with sepsis exhibit significantly elevated circulating lumican levels during the acute phase of the disease, whereas lumican-deficient mice exhibit impaired bacterial clearance and delayed recovery from sepsis [34,35,36]. The protective effects of lumican in sepsis have been attributed to its ability to boost TLR4–mediated innate immune responses and facilitate bacterial phagocytosis through interaction with the CD14 receptor via an N-terminal tyrosine residue [34,35,36]. Lumican regulates inflammation through interactions with cell-surface innate immune receptors, coreceptors, and adaptor molecules, thereby modulating the balance between proinflammatory and anti-inflammatory pathways. This regulatory function determines whether inflammation resolves appropriately or progresses to chronic fibrosis due to dysregulated tissue repair [10].

A prospective study involving patients with ARDS receiving MV reported that pulmonary lumican levels were elevated during the early phase of ARDS and were associated with pulmonary inflammation, profibrotic activity, and disease severity [9]. However, a clinical study revealed differences in lung ECM composition across different stages of diffuse alveolar damage (DAD) in patients with ARDS. The proportional area positive for lumican staining was lower in advanced-stage DAD than in early-stage DAD. Similarly, lower lumican levels were observed in lung tissue from patients with ARDS of longer duration (>7 days) than in those with ARDS of shorter duration (<7 days) [37]. These findings are consistent with our results, which revealed significantly lower levels of lumican expression in the prolonged LPS group (mice euthanized at 7 days after intratracheal LPS administration) and advanced LPS + MV group than in the control group (Figure 4). Together, the clinical findings and our experimental results suggest that dynamic changes in lumican expression contribute to the pathophysiology of ALI during disease progression. Lumican may play distinct roles during different stages of ALI. During the acute phase, increased lumican expression may facilitate tissue repair, immune cell recruitment, immune regulation, and collagen integrity maintenance. By contrast, reduced lumican expression during advanced or prolonged ALI may be associated with impaired ECM repair and progressive fibrosis. Evidence indicates that matrix metalloproteinases can degrade lumican, thereby disrupting collagen homeostasis and ECM integrity. Such disruption may contribute to the progression of fibrosis during chronic inflammation. Fibroblasts that proliferate during the early fibroproliferative phase may subsequently undergo apoptosis or exhibit reduced synthetic activity under conditions of chronic inflammation, hypoxia, and oxidative stress. This decline in fibroblast function may lead to reduced lumican production, impaired ECM repair, persistent fibrosis, and progressive lung function deterioration.

This study has several limitations. First, although prior studies have suggested that lumikine interacts with TGF-βR1/ALK5, the precise mechanisms by which lumikine stabilizes lumican and how altered lumican levels subsequently attenuates TGF-β-driven EMT and fibrogenesis remain to be elucidated. The primary aim of our study was to establish in vivo relevance and provide a rationale for future mechanistic investigations required to delineate the lumikine–lumican-TGF-β axis in fibroproliferative disease. Second, although lumican localized mainly to airway epithelial cells, other cell types, including fibroblasts, macrophages, and endothelial cells, may contributes, and cell type-specific approaches are needed to determine the dominant source of lumican among different cell populations. Third, we examined only at a single time point (8 h of MV following 7 days of LPS), whereas a time-course experiment with earlier and later MV durations would better provide stronger mechanistic insight into lumican regulation. Fourth, lumikine was administered prophylactically before LPS challenge; a rescue arm with delayed lumikine dosing after LPS would strengthen translational relevance. Finally, only male mice were studied for decreasing the effects of estrous cycles in female mice [5]. Sex hormones influence pulmonary inflammation, as well as fibrosis, chemoreflex adaption, and recovery after ALI [38]; further work should address whether male and female mice differ in susceptibility to VILI.

## 4. Materials and Methods

### 4.1. Ventilator Protocol

We used our established murine model of VILI, as previously described [5,7]. Briefly, a 20-gauge angiocatheter was applied to the tracheotomy orifice of the mouse. General anesthesia was mediated intraperitoneally with xylazine (5 mg/kg) and zoletil 50 (5 mg/kg) at the beginning of the experiment, followed by the maintenance intraperitoneal administration of zoletil 50 (5 mg/kg) and xylazine (1 mg/kg) every 30 min. Mice were positioned supine on a heating blanket and connected to a Harvard Apparatus ventilator (model 55-7058, Harvard Apparatus, Holliston, MA, USA). Ventilation was set to deliver a tidal volume of 10 mL/kg at 100 breaths per minute for 8 h, using room air with zero end-expiratory pressure. Non-ventilated control mice were anesthetized and sacrificed immediately. 

### 4.2. Lipopolysaccharide Administration

C57BL/6 male mice, 20–25 g, age 6–8 wks, received either 5 mg/kg of Salmonella typhosa lipopolysaccharide (Lot 81H4018; Sigma Chemical Co., St. Louis, MO, USA) or an equivalent volume of normal saline intratracheally for 7 days. After 1 week of spontaneous respiration to permit for development of fibrosis, the mouse will be subjected to MV for 8 h [1,2].

### 4.3. Pharmacological Reagents

Lum C13C-A (lumikine, New England Peptide, Gardner, MA, USA) 1.5 mg/kg was administered intraperitoneally 1 h before LPS management based on our current and previous studies that exhibited 1.5 mg/kg lumikine significantly attenuated the reduction in lumican expression (Figure S1) [13].

### 4.4. Statistical Evaluation

Western blot signals were quantified using ImageJ software (version 1.27z; National Institutes of Health, Bethesda, MD, USA) and expressed in arbitrary units. Data are presented as mean ± SD from at least five independent experiments. Measurements of protein oxidation, MDA, SOD activity, histopathology, and oxygenation were analyzed with StatView 5.0 (Abacus Concepts Inc., Cary, NC, USA; SAS Institute, Cary, NC, USA). All Western blot results were normalized to non-ventilated wild-type control mice breathing room air. Statistical significance was assessed by ANOVA followed by Scheffé’s post hoc test, with *p* < 0.05 considered significant.

Analysis of lung Masson’s trichrome stain histology and fibrosis scoring, and animal micro-CT, total collagen assay, western immunoblot analysis and immunohistochemical stain for lumican, EBD analysis, immunofluorescence labeling of ZO-1 and α-SMA, measurement of oxidative and antioxidant biomarkers, and MMP-9 and TGF-beta1 expression were performed as previously described [33,39]. All histopathological and immunofluorescence assessment, including fibrosis scoring, α-SMA quantification, and lumican-positive cell counts, were conducted with observers blinded to group allocation. Blinding was maintained throughout data acquisition and scoring to minimize bias.

## 5. Conclusions

The present study suggests that lumikine suppresses oxidative stress, inflammation, EMT, and total collagen accumulation, thereby attenuating biomolecular, radiological, and histopathological abnormalities by modulating the lumican pathway. Lumican is not merely a structural component of the ECM; it also actively participates in the transition from inflammatory ALI to pulmonary fibrogenesis. Understanding the dual roles of lumican in inflammation and fibrogenesis in our two-hit animal model of endotoxin exposure and mechanical stretch may help researchers comprehend the molecular mechanisms underlying the pathogenesis of ALI associated with sepsis and MV. The finding that lumikine ameliorates VILI in endotoxemic mice may inform ALI management strategies for patients with sepsis requiring MV.

## Figures and Tables

**Figure 1:**
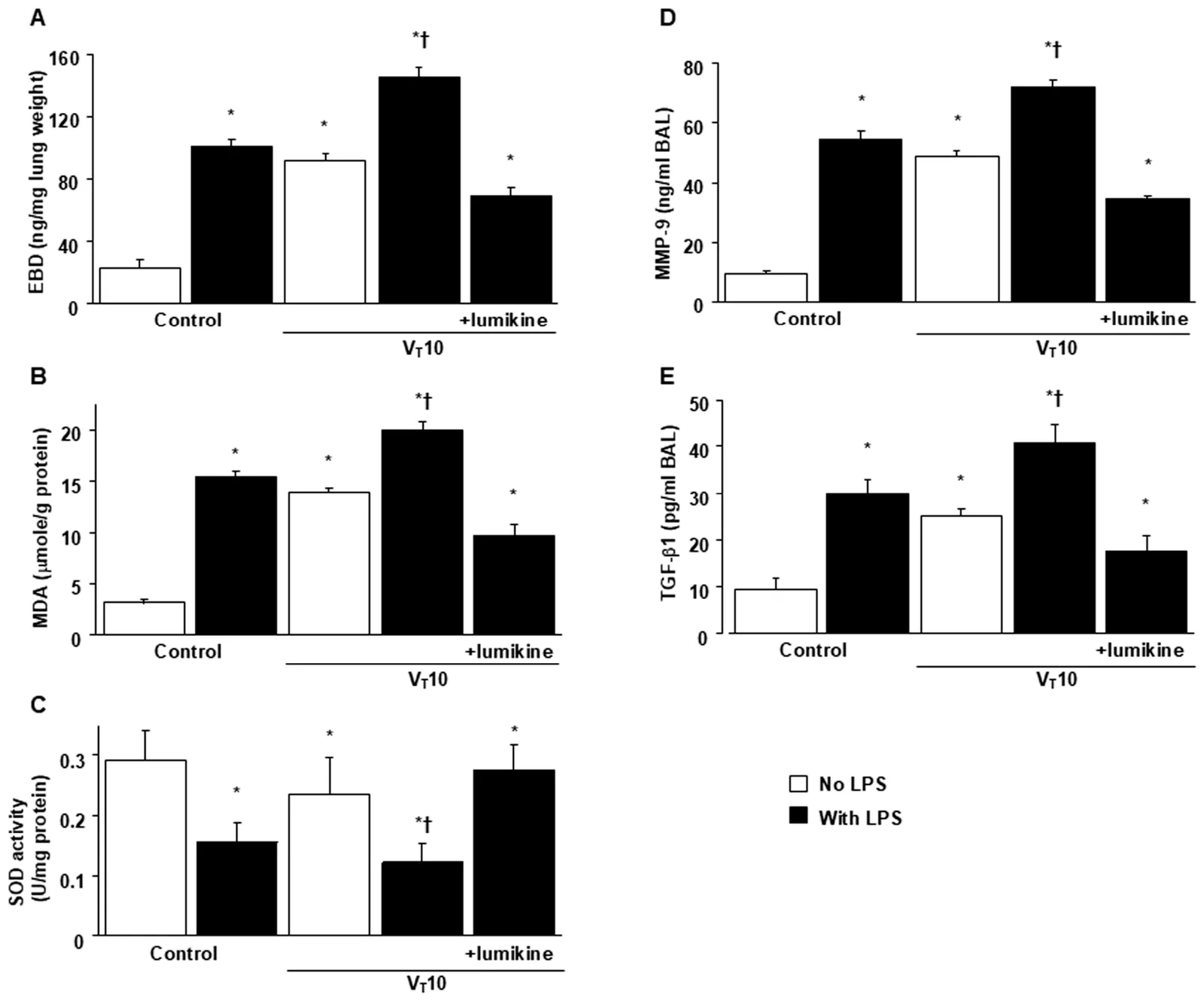
Reduction in endotoxin-enhanced lung stretch-induced lung injury by lumikine. (**A**) Evans blue dye analysis, (**B**) MDA, (**C**) SOD, (**D**) MMP-9, and (**E**) TGF-β1 secretion in BAL fluid were from the lungs of nonventilated control mice and mice ventilated at a tidal volume (V_T_) of 10 mL/kg for 8 h with or without LPS administration (*n* = 5 per group). Lumikine 1.5 mg/kg was given intraperitoneally 1 h before LPS management. * *p* < 0.05 versus the nonventilated control mice without LPS; † *p* < 0.05 versus all other groups. BAL = bronchoalveolar lavage; EBD = Evans blue dye; LPS = lipopolysaccharide; MDA = malondialdehyde; MMP-9 = metalloproteinase-9; SOD = superoxide dismutase; TGF-β1 = transforming growth factor-β1.

**Figure 2:**
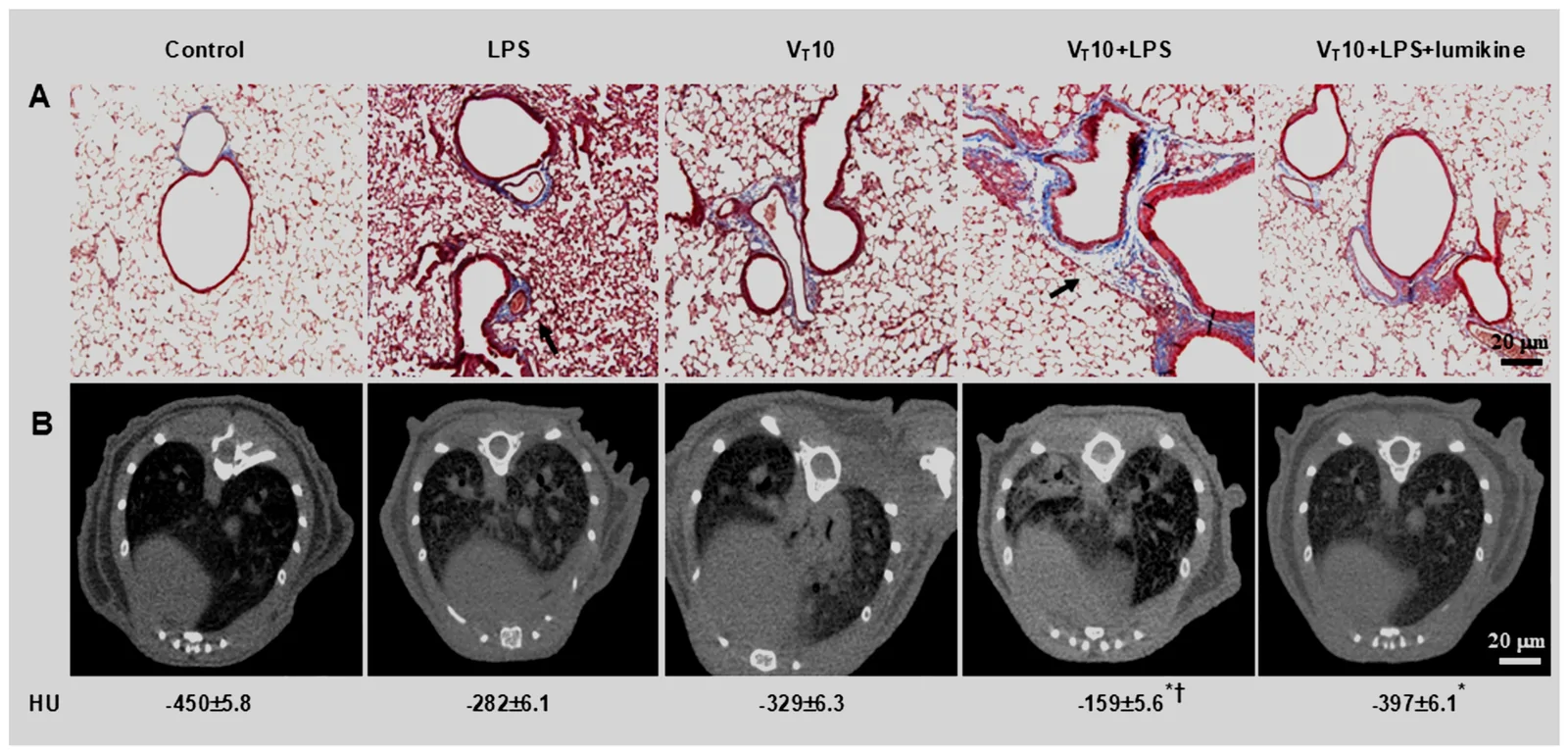
Reduction in lung stretch-induced collagen production by lumikine. (**A**) Representative micrographs (×100) with Masson’s trichrome staining of paraffin lung sections (*n* = 5 per group) of the lung sections, and (**B**) micro-computed tomography imaging of lung tissue were from the lungs of nonventilated control mice and mice ventilated at a tidal volume of 10 mL/kg for 8 h with or without LPS administration (*n* = 5 per group). For each mouse, the 10 axial CT slices encompassing the largest total lung area were selected, and the Hounsfield unit values from these slices were averaged to obtain a per-animal mean. The group mean was calculated from the averaged values of all animals. Lumikine 1.5 mg/kg was given intraperitoneally 1 h before LPS management. * *p* < 0.05 versus the nonventilated control mice without LPS; † *p* < 0.05 versus all other groups. Scale bars represent 20 µm. HU = Hounsfield unit.

**Figure 3:**
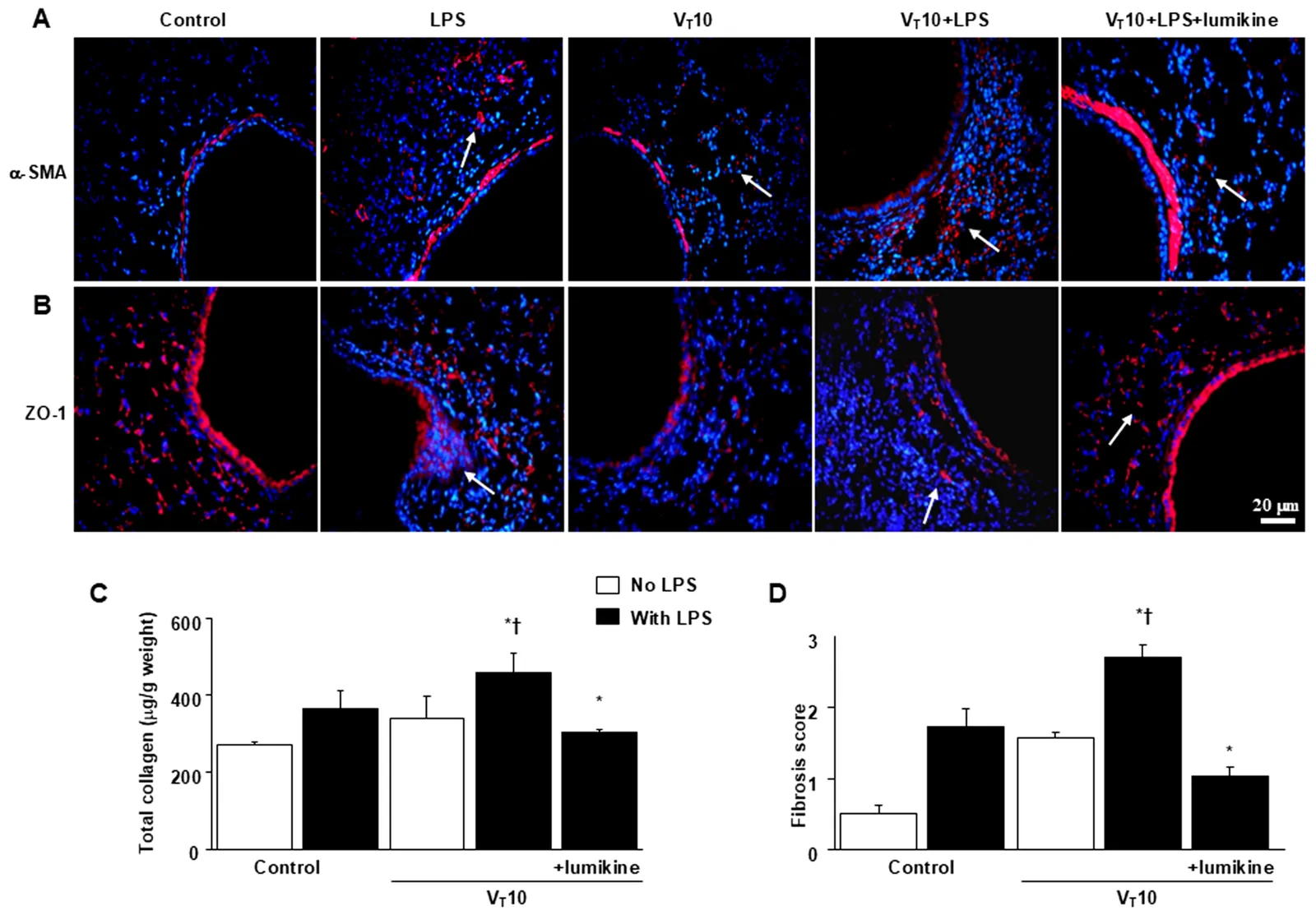
Suppression of lung stretch-induced fibrogenic markers by lumikine. (**A**,**B**) Representative photomicrographs (×400) with α-smooth muscle actin (α-SMA, red), Zonula occludens (ZO-1, red), and Hoechst (blue) immunofluorescent staining of paraffin lung sections (*n* = 5 per group) were from the lungs of nonventilated control mice and mice ventilated at a tidal volume of 10 mL/kg for 8 h with or without LPS administration (*n* = 5 per group). Positive red staining in the lung epithelium and interstitium is identified by arrows (*n* = 5 per group). (**C**) Total lung collagen content was from the lungs of nonventilated control mice and mice ventilated at a tidal volume of 10 mL/kg for 8 h with or without LPS administration (*n* = 5 per group). (**D**) The fibrotic scoring was quantified as the average number of 10 nonoverlapping fields in Masson’s trichrome staining of paraffin lung sections (*n* = 5 per group). Lumikine 1.5 mg/kg was given intraperitoneally 1 h before LPS management. Scale bars represent 20 μm. * *p* < 0.05 versus the nonventilated control mice without LPS pretreatment; † *p* < 0.05 versus all other groups.

**Figure 4:**
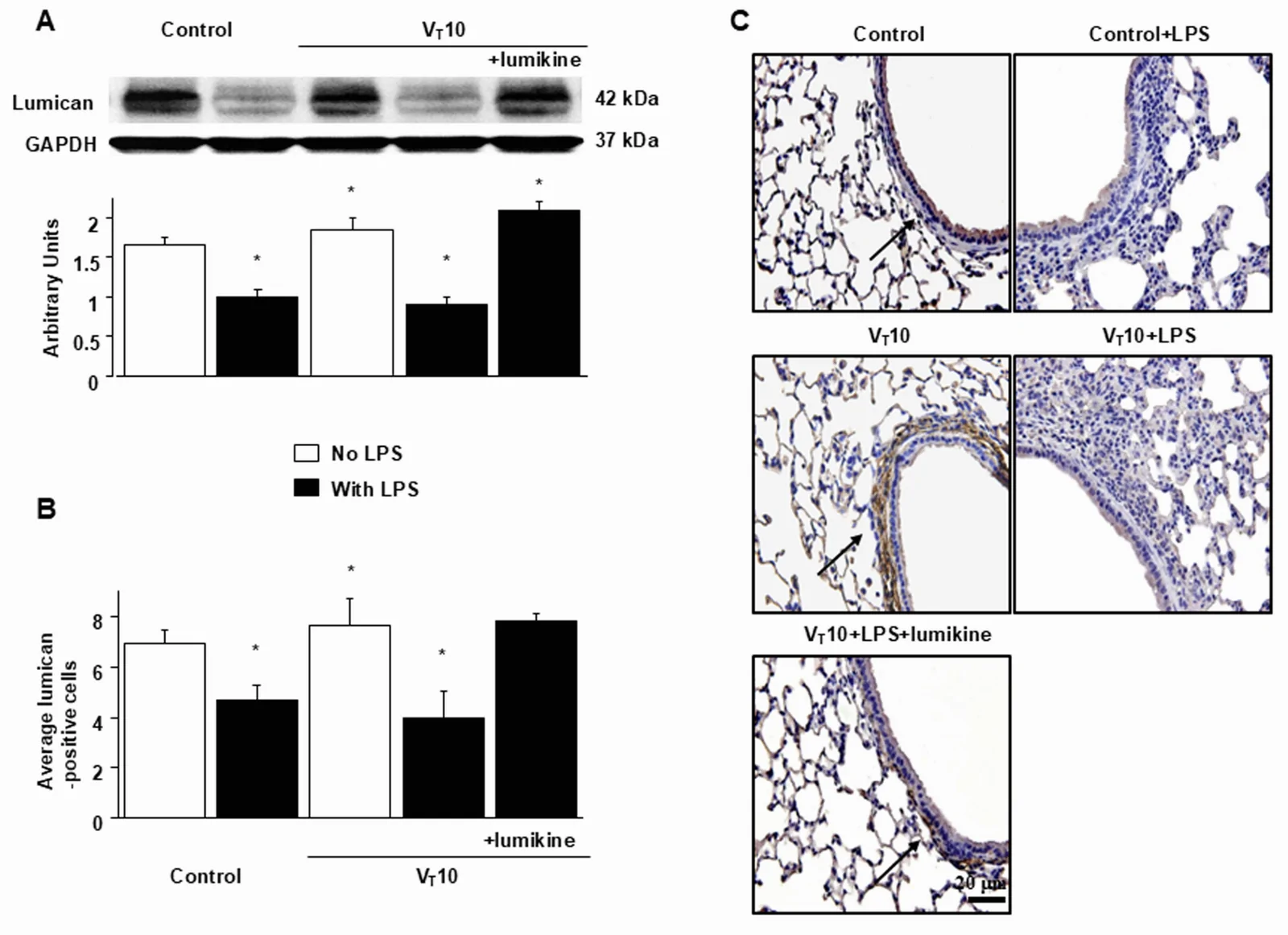
Reduction in endotoxin-augmented lung stretch-induced lumican protein expression by lumikine. (**A**) Western blots were performed using antibodies that recognize lumican and GAPDH expression in lung tissues from nonventilated control mice and mice ventilated at a tidal volume of 10 mL/kg for 8 h with or without LPS administration (*n* = 5 per group). Arbitrary units were expressed as the ratio of relative lumican activation (*n* = 5 per group). (**B**,**C**) Representative micrographs (×400) with lumican staining of paraffin lung sections and quantification were from nonventilated control mice and mice ventilated at a tidal volume of 10 mL/kg for 8 h with or without LPS administration (*n* = 5 per group). Lumikine 1.5 mg/kg was given intraperitoneally 1 h before LPS management. A dark-brown diaminobenzidine signal identified by arrows indicates positive staining for lumican in the lung epithelium or interstitial, whereas shades of bluish tan signify nonreactive cells. Scale bars represent 20 μm. * *p* < 0.05 versus the nonventilated control mice without LPS; GAPDH = glyceraldehyde–phosphate dehydrogenase.

**Figure 5:**
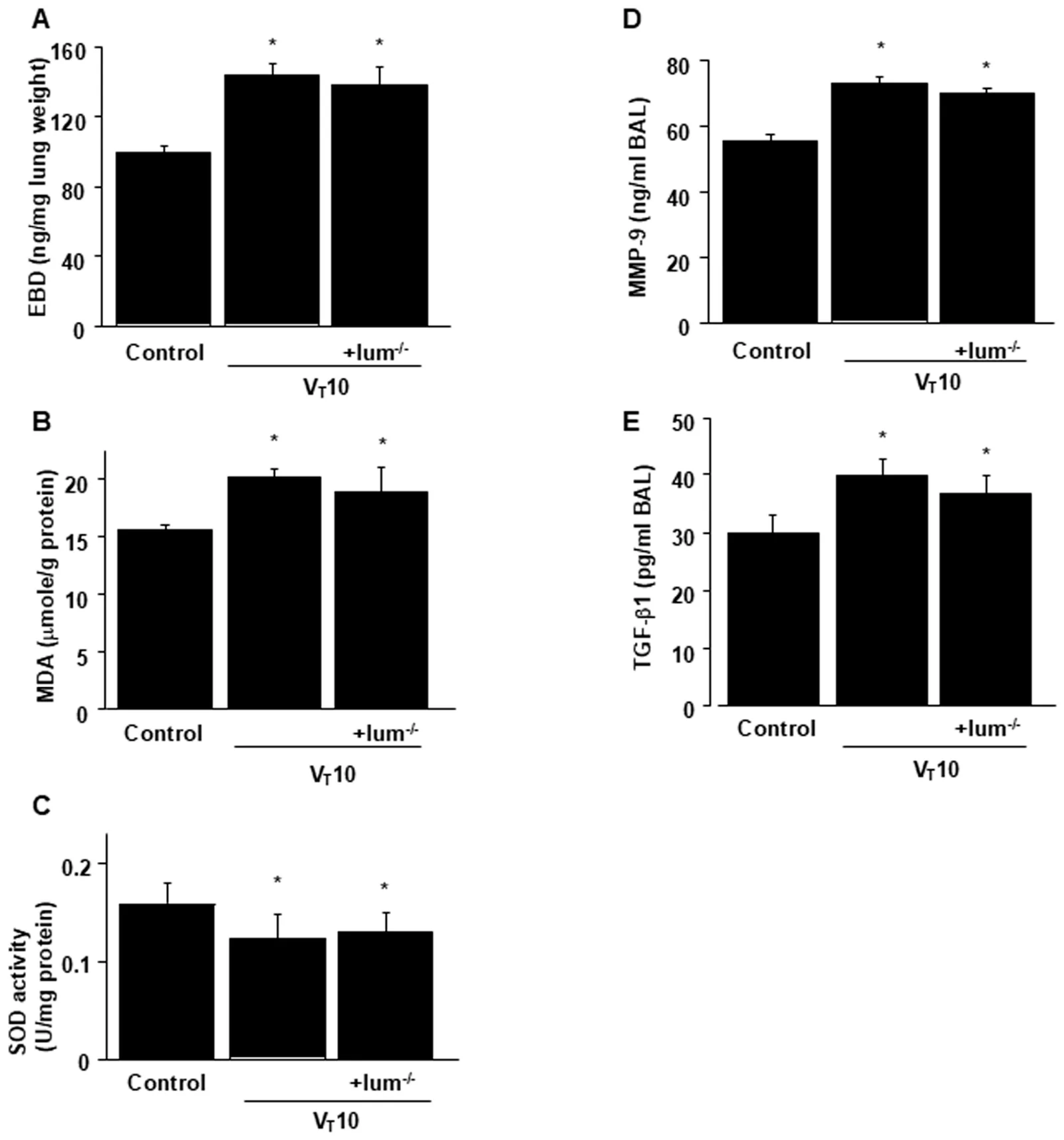
Effects of endotoxin-enhanced lung stretch-induced lung injury in lumican-deficient mice. (**A**) Evans blue dye analysis, (**B**) MDA, (**C**) SOD, (**D**) MMP-9, and (**E**) TGF-β1 secretion in BAL fluid were from the lungs of nonventilated control mice and mice ventilated at a tidal volume of 10 mL/kg for 8 h with LPS administration (*n* = 5 per group). * *p* < 0.05 versus the nonventilated control mice with LPS.

**Figure 6:**
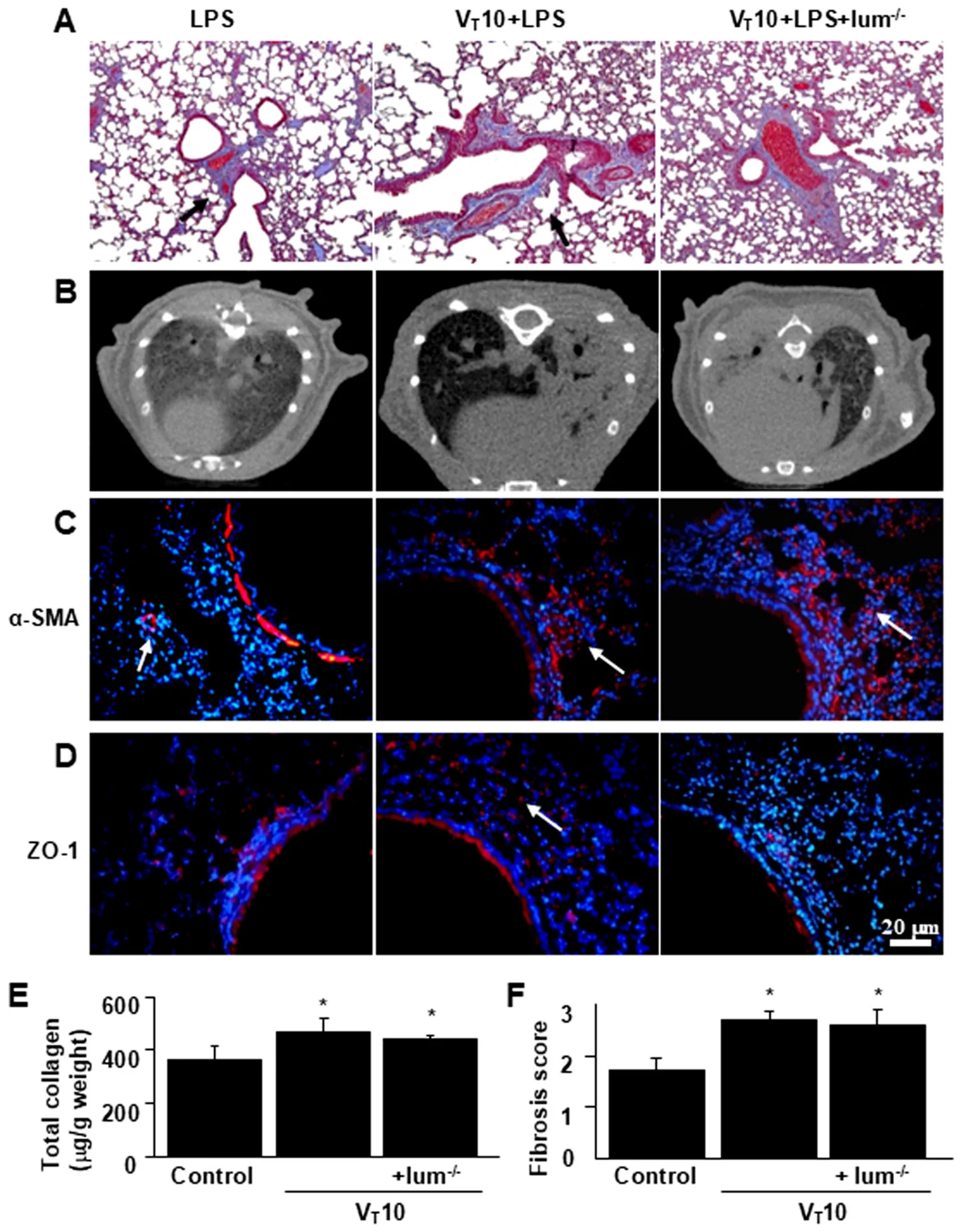
Effects of lung stretch-induced collagen production and fibrogenic markers in lumican-deficient mice. (**A**) Representative micrographs (×100) with Masson’s trichrome staining of paraffin lung sections (*n* = 5 per group) of the lung sections, and (**B**) micro-computed tomography imaging (*n* = 5 per group) of lung tissue were from the lungs of nonventilated control mice and mice ventilated at a tidal volume of 10 mL/kg for 8 h with LPS administration (*n* = 5 per group). (**C**,**D**) Representative photomicrographs (×400) with α-smooth muscle actin (α-SMA, red), Zonula occludens (ZO-1, red), and Hoechst (blue) immunofluorescent staining of paraffin lung sections (*n* = 5 per group) were from the lungs of nonventilated control mice and mice ventilated at a tidal volume of 10 mL/kg for 8 h with or without LPS administration (*n* = 5 per group). Positive red staining in the lung epithelium and interstitium is identified by arrows (*n* = 5 per group). (**E**) Total lung collagen content was from the lungs of nonventilated control mice and mice ventilated at a tidal volume of 10 mL/kg for 8 h with or without LPS administration (*n* = 5 per group). (**F**) The fibrotic scoring was quantified as the average number of 10 nonoverlapping fields in Masson’s trichrome staining of paraffin lung sections (*n* = 5 per group). * *p* < 0.05 versus the nonventilated control mice with LPS.

**Figure 7:**
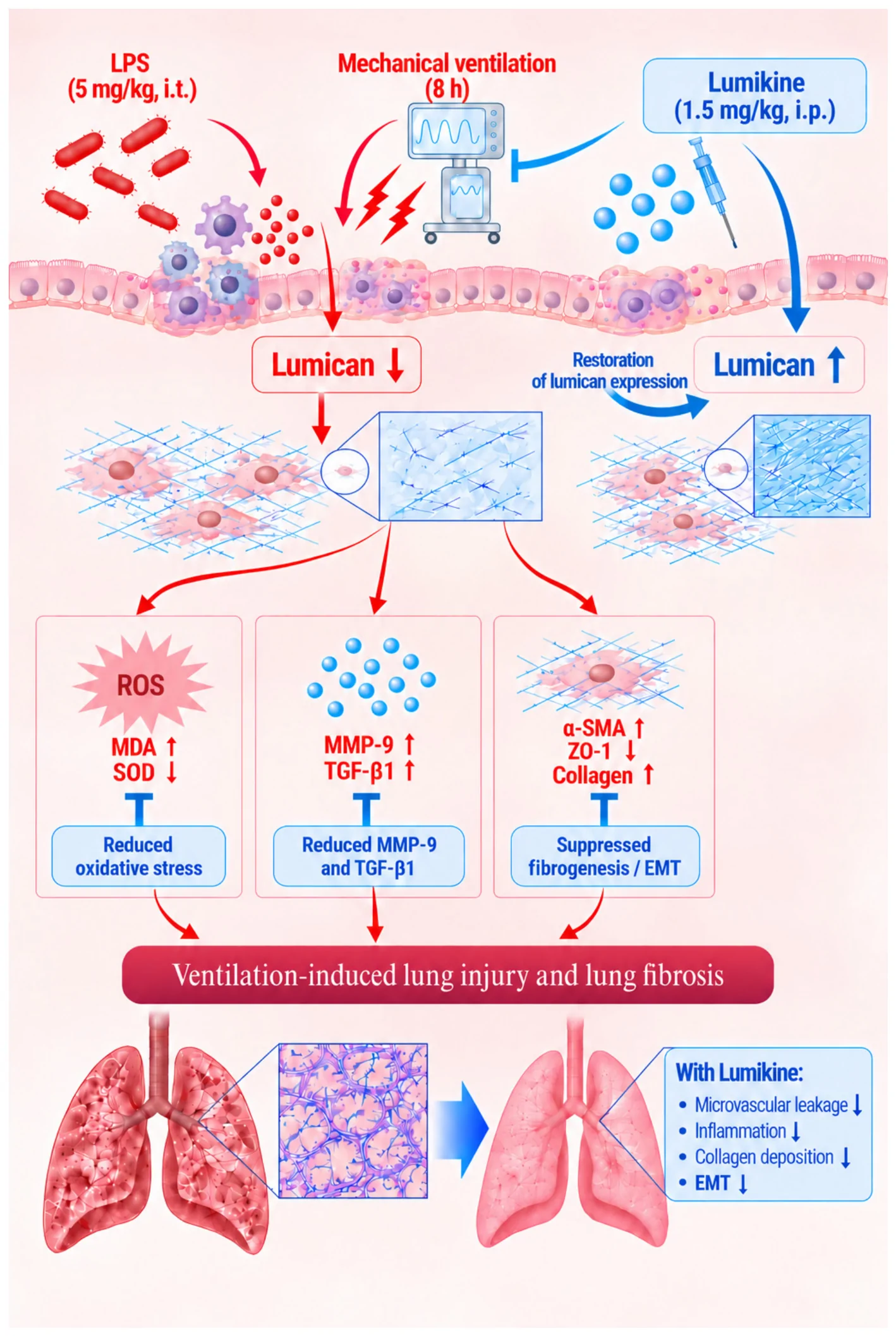
Schematic figure illustrating the signaling pathway activation with mechanical ventilation and endotoxemia. Endotoxin-induced augmentation of mechanical stretch-mediated cytokine production and lung fibrosis were attenuated by pharmacological inhibition with lumikine. LPS = lipopolysaccharide; MDA = malondialdehyde; MMP-9 = metalloproteinase-9; ROS = reactive oxygen species; α-SMA = α-smooth muscle actin; SOD = superoxide dismutase; TGF-β1 = transforming growth factor-β1; VILI = ventilator-induced lung injury; ZO-1 = Zonula occludens.

## Data Availability

The data presented in this study are available on request from the corresponding author.

## Supplementary Materials

The following supporting information can be downloaded at: https://www.mdpi.com/article/10.3390/ijms27198515/s1.

## Abbreviations

The following abbreviations are used in this manuscript:

LPS	Lipopolysaccharide
MV	Mechanical ventilation
ALI	Acute lung injury
ECM	Extracellular matrix
VILI	Ventilator-induced lung injury
MMP-9	Matrix metalloproteinase-9
TGF-β1	Transforming growth factor-β1
Micro-CT	Micro-computed tomography
ZO-1	Zonula occludens-1
α-SMA	α-smooth muscle actin
EMT	Epithelial-to-mesenchymal transition
ARDS	Acute respiratory distress syndrome
Lum^−/−^	Lumican-deficient
DAD	Diffuse alveolar damage
BAL	Bronchoalveolar lavage
EBD	Evans blue dye
MDA	Malondialdehyde
SOD	Superoxide dismutase
ROS	Reactive oxygen species
HU	Hounsfield unit
