# Supplementary material for: Lumikine Attenuates Ventilation-Induced Lung Injury in Mice with Lipopolysaccharide-Induced Lung Fibrosis
Source: Int J Mol Sci. 2026 Sep 24;27(19):8515. doi: 10.3390/ijms27198515 (PMC13665356; doi:10.3390/ijms27198515)
Supplement: Supplementary file 1 [file ijms-27-08515-s001.zip › Fig S1.pdf]

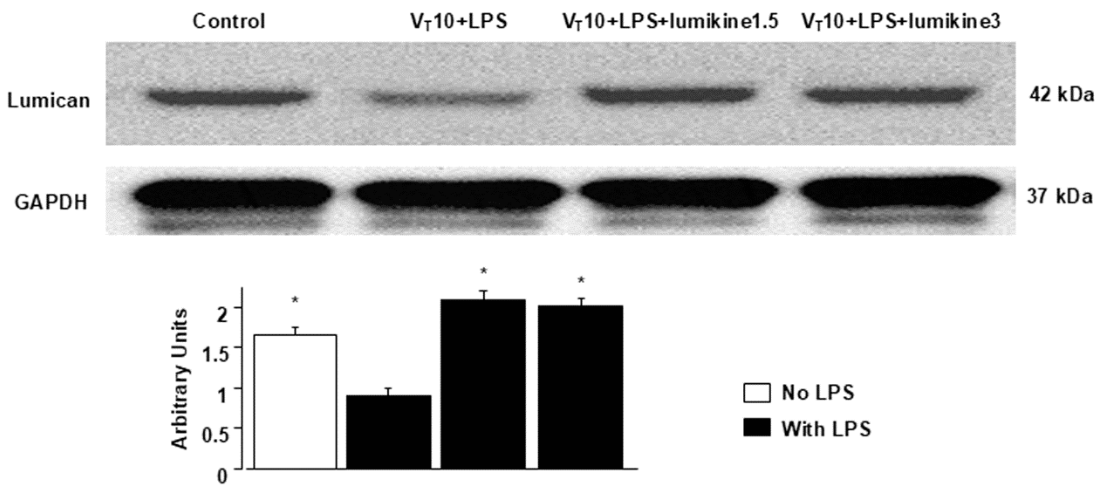

Figure S1. Western blots were performed using antibodies that recognize lumican and GAPDH expression in lung tissues from nonventilated control mice and mice ventilated at a tidal volume (V_T) of 10 mL/kg for 8 h with or without LPS administration (n = 5 per group). Different doses of lumikine (1.5 mg/kg and 3 mg/kg) were demonstrated to substantially attenuate the reduction in lumican expression observed in mice treated with LPS and MV. No significant differences were observed between 1.5 mg/kg and 3 mg/kg lumikine administration, thus 1.5 mg/kg was chosen in this experiment. Arbitrary units were expressed as the ratio of relative lumican activation (n = 5 per group). * $P < 0.05$ versus the mice ventilated at a tidal volume of 10 mL/kg for 8 h with LPS administration. LPS = lipopolysaccharide.
