# Supplementary material for: Lumikine Attenuates Ventilation-Induced Lung Injury in Mice with Lipopolysaccharide-Induced Lung Fibrosis
Source: Int J Mol Sci. 2026 Sep 24;27(19):8515. doi: 10.3390/ijms27198515 (PMC13665356; doi:10.3390/ijms27198515)
Supplement: Supplementary file 1 [file ijms-27-08515-s001.zip › Li LF. Lumikine manuscript table S1.pdf]

Table S1. Physiologic conditions at the beginning and end of ventilation.

	Nonventilated	Nonventilated LPS	V_T 10 ml/kg	V_T 10 ml/kg LPS	V_T 10 ml/kg LPS+ lumikine	V_T 10 ml/kg LPS+ $Lum^{-/-}$
PH	7.41±0.04	7.38±0.02	7.36±0.07	7.37±0.04	7.37±0.06	7.39±0.04
PaO2 (mmHg)	98.5±0.2	79.1±0.2	84.6±3.2*	75.1±2.8*	88.5±2.3*	80.3±1.9*
PaCO2 (mmHg)	39.1±0.3	38.6±0.3	38.4±1.6	38.5±1.4	37.8±1.2	37.6±1.5
MAP (mmHg)						
Start	85.6±1.2	83.1±0.5	82.7±2.5	82.4±2.7	84.7±2.1	83.6±2.4
End	85.1±0.4	76.2±0.4	75.7±2.1*	74.9±2.1*	78.3±2.4*	76.1±2.2*
PIP (mmHg)						
Start			16.3±1.2	16.1±1.5	15.9±1.2	15.9±1.3
End			17.4±1.8	17.6±1.8	17.2±1.4	17.3±1.4

At the end of the study period, we obtained data of mean arterial pressure and arterial blood gases from the nonventilated control mice and mice ventilated at a tidal volume of 10 ml/kg for 8 h (n = 5 per group). The normovolemic statuses of mice were maintained by monitoring mean artery pressure. Data are presented as means ± SDs. * Indicates that $P < 0.05$ when compared to the nonventilated control mice with LPS. LPS = lipopolysaccharide; MAP = mean arterial pressure; PIP = peak inspiratory pressure; V_T = tidal volume.
